# Critical analysis for nonlinear oscillations by least square HPM

**DOI:** 10.1038/s41598-024-51706-3

**Published:** 2024-01-17

**Authors:** Muhammad Rafiq, Muhammad Kamran, Hijaz Ahmad, Afis Saliu

**Affiliations:** 1 Department of Mathematics, COMSATS University Islamabad, Wah Campus, Islamabad, Pakistan; 2 Department of Mathematics, Faculty of Science, Islamic University of Madinah, Madinah, Saudi Arabia; 3 Near East University, Operational Research Center in Healthcare, TRNC Mersin 10, Nicosia, 99138, Turkey; 4 Center for Applied Mathematics and Bioinformatics, Gulf University for Science and Technology, Mishref,, Kuwait; 5 Department of Computer Science and Mathematics, Lebanese American University, Beirut, Lebanon; 6 Department of Mathematics, University of the Gambia, MDI Road, P.O. Box 3530, Kanifing, Serrekunda, The Gambia

**Keywords:** Mathematics and computing, Physics

## Abstract

In this study, a novel adapted homotopy perturbation method (HPM) is used to treat the nonlinear phenomena of free vibration in a system with one degree of freedom. This adaptation involves the integration of HPM with a least-squares optimizer, resulting in a hybrid method called the least square homotopy perturbation method (LSHPM). The LSHPM is tested on various nonlinear problems documented in the existing literature. To evaluate the effectiveness of the proposed approach, the identified problems are also tackled using HPM and the MATLAB built-in function bvp5c, and then the results are compared with those obtained using LSHPM. In addition, a comparative analysis is carried out with the results of the AG method as found in the literature. The results show that LSHPM is a reliable and efficient method suitable for solving more complicated initial value problems in the fields of science and engineering.

## Introduction

An oscillation system denotes a dynamic system that demonstrates periodic motion around an equilibrium point. Oscillations are ubiquitous in the natural world, appearing in various physical, mechanical, electrical, and biological systems. A comprehensive understanding of oscillation principles holds paramount importance in disciplines such as physics, engineering, and biology.

Dynamic systems that manifest oscillations or vibrations departing from linearity are referred to as nonlinear oscillating systems.These systems are commonly characterized by nonlinear differential equations, capturing intricate and at times unpredictable dynamics. These kinds of nonlinear oscillatory systems are found in many different scientific and engineering fields, including mechanics^1^, electromagnetic phenomena^2^, fluid dynamics^3^, biology^4–7^, chemistry^8^, and other fields^9^, including particle physics and cosmology^10^. These systems describe several applications of nonlinear oscillating dynamics. A few examples of this diversity are as follows: an electronic circuit that integrates nonlinear components such as diodes or transistors^11^; a pendulum that exhibits significant amplitude swings and displays nonlinear characteristics^12^; a chemical reaction that displays oscillatory tendencies due to nonlinear kinetics^13^; and animal populations that oscillate due to nonlinear interactions^14^.

One of the main features of nonlinear oscillatory systems is their sensitivity to initial conditions and parameters. Minor adjustments to these parameters may cause the system to function significantly in a different way, which may lead to the rise of disordered dynamics or other oscillatory types^15^. Despite their intricacy, nonlinear oscillatory systems are an vital area of investigation that suggest critical perceptions into a class of real and synthetic systems. Through in-depth research and modelling, scientists and engineers can learn a deep knowledge of the main procedures of these systems, which they can then use to develop the functionality and shape of various systems. Nonlinear oscillators are currently being intensively explored in a wide range of study disciplines, including as multi-body systems, vibrations, transportation, structural dynamics, and others^16–18^.

Various methods, such as He’s Energy Balance method^19,20^, the Max-Min methodology^2^, and the homotopy analysis method^21^, have been introduced by researchers to tackle the challenges presented by nonlinear differential equations in oscillatory systems. These methodologies provide effective approaches for analyzing and predicting the behavior of intricate systems.

In 2012, Ganji and Azimi^22^ addressed specific nonlinear oscillation systems by applying the max-min technique and developing an amplitude-frequency formulation. In 2022, Samadi et al.^16^ tackled similar problems utilizing the AGM and HPM procedures, and they compared their results with those obtained through the RK4 technique. Qie et al.^23^ introduced a straightforward and distinctive method for addressing highly nonlinear oscillators. Their study offers an efficient approach to rapidly establish the amplitude-frequency correlation of a nonlinear oscillator. The investigation conducted by Mohammadian and colleagues^24^ focused on examining the applicability of the AGM and its enhancement for nonlinear damped oscillators. In 2021, El-Dib^25^ utilized the HPM technique in conjunction with a rank-upgrading approach to achieve superior results in nonlinear oscillation.

Oscillation systems, commonly encountered in various scientific and engineering disciplines, are frequently characterized and examined using mathematical models. These models serve as reliable instruments for predicting, understanding, and controlling the dynamic features of oscillatory processes. Researchers and engineers can find notable understandings into the complexities of oscillation systems by explaining physical principles into mathematical formulas.

Oscillation systems, distinguished by their rhythmic and repetitive motion, have a close connection with the language of differential equations. This association between oscillations and differential equations offers a robust framework for articulating and comprehending the dynamic characteristics of these systems across various scientific disciplines.

Differential equations serve as a modeling tool for problems involving diverse independent variables^26–28^. This area of study holds significance in mathematics, addressing intriguing issues related to the modeling of various phenomena in physics, biology^29–31^, and engineering^32,33^. For instance, phenomena like unsteady squeezing flow of heat and mass transfer^34^ and MHD Boundary Layer Flow over a Stretching Sheet^26^ find representation through differential equations, as the rate of change is a fundamental expression for describing physical phenomena in scientific investigations^35^. Diverse analytical and numerical methodologies are employed to gain valuable insights into system behavior, unraveling complexities inherent in the study. These approaches encompass the homotopy analysis method^36–38^, optimal homotopy asymptotic method^39–41^, Adomian decomposition method^42^, extended optimal homotopy asymptotic method^39^, the coupling of Runge-Kutta methods with the MATLAB neural network built-in function nftool^40,43^, the conjunction of the homotopy analysis method with the neural network MATLAB function nftool^40,44^, utilization of artificial neural networks^45^, the coupling of the MATLAB built-in function nftool with the homotopy asymptotic method^46^, and the combination of the Levenberg-Marquardt technique with the MATLAB neural network nftool^35,47^.

The HPM has been extensively examined since 1999, evolving into a valuable mathematical tool due to the collaborative efforts of numerous scientists. Researchers have validated the convergence of the ground breaking HPM across various scenarios^48^, and diverse modifications have surfaced in the scholarly literature. Upon using the phrase “modified homotopy perturbation method” as a search query in Clarivate Analytics’ Web of Science, we identified over 400 relevant items. The combination of HPM with other methods has garnered significant attention, such as the Generalized Differential Quadrature Method^49^ and the Fourier transform^50^. The LSHPM, a hybrid of the HPM and the least square method^51,52^. It is Remarkably effective for solving ordinary, partial, and fractional differential equations

In 2012 GANJI1 and AZIMI^22^ applied the Max-Min Approach(MMA) and Amplitude Frequency Formulation (AFF) to derive the approximate analytical solution for motion of nonlinear free vibration of conservative, single degree of freedom systems. The results were compared with the results obtained by forth-order runge-kutta method. In 2022 Sanadi et al.^16^ used the Akbari Ganji Method, abbreviated as AGM, and the HPM, to analytically investigate some oscillating systems with nonlinear behavior in a variety of situations and compare the results to the numerical approach to assess the validity and accuracy of these methods. The findings in all situations showed the precision of both the AGM and HPM, with all computations showing an amazing similarity to the RK4 method. In this manuscript, we use the newly developed LSHPM and MATLAB builtin functiobn bvp5c to unfold the problem^22^. This method combines HPM with a least squares minimization step, which minimizes approximation errors and speeds up convergence, therefore increasing the method’s effectiveness. Compared to other iterative techniques, it provides accurate solutions with fewer iterations. LSHPM is a priceless instrument in a variety of scientific and technical fields due to its many advantages, which include simplicity in using, accuracy, and computational efficacy.

## Methodology

We use the newly developed LSHPM and the bvp5c function integrated in MATLAB to solve the problem.

### LSHPM

To understand the main idea about the LSHPM, let’s examine the following nonlinear differential equation

1 $$\begin{aligned} A(v) -g(\xi )=0, \ \ \xi \in \Omega , \end{aligned}$$

with the boundary conditions

2 $$\begin{aligned} B(v, \frac{\partial v}{\partial n})=0, \ \ \xi \in \Gamma , \end{aligned}$$

Equation (1) can be expressed in the following format: *A* denotes a differential operator of general type, *B* represents a boundary operator, *g* is a well-defined analytic function and $\Gamma$ denotes the boundary surrounding the domain $\Omega$. We have further divided *A* into its linear and nonlinear summands, *L* and *N*, respectively. Consequently, we can reformulate the Eq. (1) as follows

3 $$\begin{aligned} L(v) + N(v)-g(\xi )=0, \ \ \xi \in \Omega . \end{aligned}$$

In the Eq. (3), our objective is to solve it using the HPM. To achieve this, we establish a homotopy function $H:{\mathbb {R}}^{n} \times [0, 1]\longrightarrow {\mathbb {R}}^{m}$, for Eq. (3), and this homotopic function *H* satisfies the convex homotopic property as given below

4 $$\begin{aligned} H(v, p) = (1-p)\left[ L(v) - L(v_{0})\right] + p\left[ L(v) + N(v) - g(\xi )\right] = 0, \quad \xi \in \Omega , \quad p \in [0, 1]. \end{aligned}$$

Here, *p* represents an embedding parameter, while $v_{0}$ is an initial approximation of Eq. (1), satisfying the boundary conditions (2). Notably, from Eq. (4), it is evident that

5 $$\begin{aligned} H(v, 0) = L(v) - L(v_{0}) = 0, \end{aligned}$$

and

6 $$\begin{aligned} H(v, 1) = L(v) + N(v) - f(\xi ) = A(v) - g(\xi ) = 0, \quad \xi \in \Omega . \end{aligned}$$

The progression of the parameter *p* from 0 to 1 corresponds to the transformation of *H*(*v*, *p*) from $v_{0}$ to *v*. The function *H*, parametrized by *p*, establishes a continuous trajectory from the known initial solution $v_{0}$ to the desired solution *v*. This function is identified as a homotopy linking the functions $H(v, 0) = L(v) - L(v_{0})$ and $H(v, 1) = A(v) - g(\xi )$. Moreover, $L(v) - L(v_{0})$ and $A(v) - g(\xi )$ are recognized as homotopic. The symbol ${\mathbb {R}}$ denotes the set of real values. As the parameter *p* varies within the interval [0, 1], $v_0$ progressively converges toward the solution *v*. It is reasonable to posit that the solution to this equation can be represented as a series involving powers of *p*, as discussed in references^53^.

7 $$\begin{aligned} v = v_{0}+pv_{1}+p^{2}v_{2}+p^{3}v_{3}+\cdots. \end{aligned}$$

Taking $p\rightarrow 1$, estimated result that HPM will produce is

8 $$\begin{aligned} {\tilde{u}} = \lim _{p\rightarrow 1} v= v_{0}+ v_{1}+v_{2}+ v_{3}+\cdots. \end{aligned}$$

After introducing the unknown constants $c's$ into the derived series solution ${\tilde{u}}$ given by Eq. (8) as coefficients of $v's$ in order to control the convergence, we designate this new series as ${\tilde{U}} = \sum _{i=0}^{\infty }c_{i}v_{i}$. Subsequently, we substitute the approximate solution ${\tilde{U}}$ in place of the unknown function *v* in Eq. (3) to construct the residual function.

9 $$\begin{aligned} {\hat{R}}(\xi , c_{i})= L({\tilde{U}} ) + N({\tilde{U}} )-g(\xi ), \ \ \xi \in \Omega .. \end{aligned}$$

We now determine the residual sum of squares

10 $$\begin{aligned} J( c_{i}) = \int _{\Omega } {\hat{R}}^{2}(\xi , c_{i})d \xi ,\ \ i=0,\ 1,\ 2,\ \cdots. \end{aligned}$$

The optimal values for the unknown constants $c_{i}$ are established by solving the system of equations $\frac{\partial J}{\partial c_{i}} = 0$. Afterward, by substituting these determined values of $c_{i}$ back into ${\tilde{U}}$, we achieve the desired solution through the application of the Least Square Homotopy Perturbation Method (LSHPM) approach.

### bvp5c

The bvp5c code utilizes a finite difference implementation based on the four-stage Lobatto IIIa formula, as described in^54^. This formula, employing collocation, produces a $C^{1}$ continuous solution with fifth-order accuracy consistently over the interval [*a*, *b*], where $a, b \in \Re$. The implementation adopts the Lobatto IIIa formula as an implicit Runge-Kutta formula. The MATLAB built-in function bvp5c is specifically crafted to directly solve the associated algebraic equations, ensuring a smooth and efficient solution process.

## Oscillating systems

**Figure 1:**
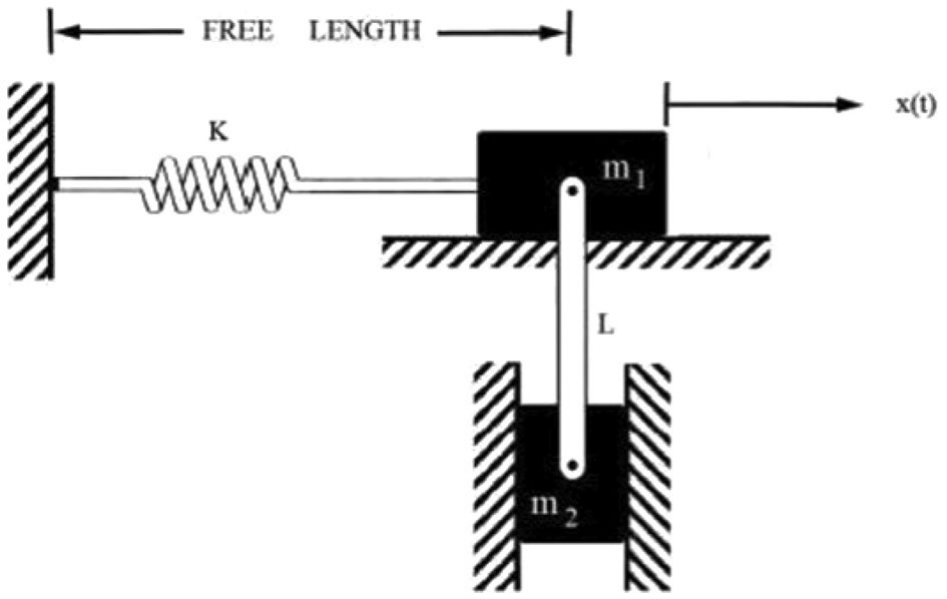
Geometric representation of Case 1.

**Case 1.** In Fig. 1, we assign $m_{1}$ as the mass of the horizontal block, and $m_{2}$ represents the mass of the vertically displaced block connected to $m_{1}$. The symbol *L* denotes the length, *g* stands for the acceleration due to gravity, and *k* represents the spring constant, as illustrated in Fig. 1. Introducing an additional parameter, denoted as *v*, defined as $\displaystyle v = \frac{x}{L}$, with the condition that $|v|<< 1$. With this definition, the differential equation can be expressed in the following manner, as detailed in references^16,22^:

11 $$\begin{aligned} \ddot{v}+\ddot{v}v^{2}\frac{m_{2}}{m_{1}}+\frac{m_{2}}{m_{1}}v {\dot{v}}^{2}+\bigg (\frac{k}{m_{1}}+\frac{gm_{2}}{Lm_{1}}\bigg )v+\frac{gm_{2}}{2Lm_{1}}v^{3}=0 \end{aligned}$$

In this context, *v*, ${\dot{v}}$, and $\ddot{v}$ represent the dimensionless displacement, velocity, and acceleration, respectively, of the vibrating system. The initial displacement and velocity are specified as follows:

12 $$\begin{aligned} v(0)=A, {\dot{v}}(0)=0. \end{aligned}$$

In the presented model, it is apparent that the restoring force of the springs follows a linear pattern. However, the introduction of damping introduces nonlinearity, leading to a mathematical model that deviates from linearity.

**Figure 2:**
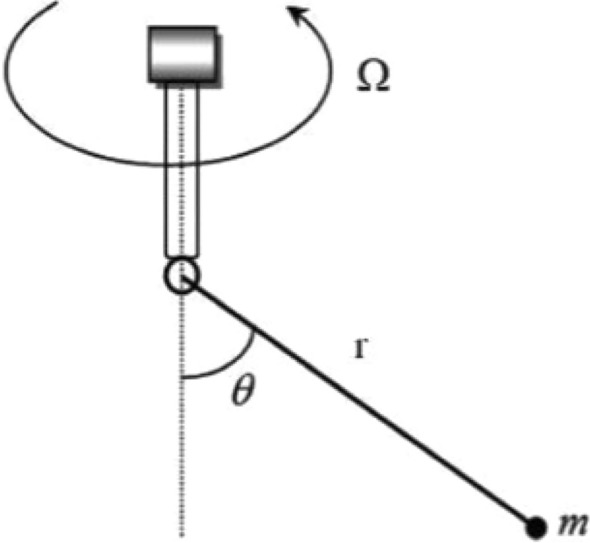
Geometric representation of Case 2.

**Case 2.** Figure 2 depicts a simple pendulum with a rod connected to a rotating rigid frame. The rigid frame experiences continuous rotation at an angular velocity represented by $\Omega$ around the vertical axis, giving rise to the formulation of the ensuing nonlinear differential equation.^16,22^,

13 $$\begin{aligned}{} & {} \frac{d^{2}\theta }{dt^{2}}+(1-\Lambda \cos \theta )\sin \theta =0, \end{aligned}$$

14 $$\begin{aligned}{} & {} \theta (0)= A, \ \ \ \frac{d\theta }{dt}|_{t=0}=0, \end{aligned}$$

where, $\displaystyle \Lambda =\frac{r\Omega ^{2}}{g},$ the variables $\theta$ and *t* are dimensionless and represent displacement and time, respectively. In the context of the Eq. (13), the expression $-\Lambda \cos \theta \sin \theta$ is a result of the movement of the rotating rigid frame.

Consider

15 $$\begin{aligned} \sin \theta =\theta -\frac{\theta ^{3}}{3!}+ \frac{\theta ^{5}}{5!}-\cdots , \end{aligned}$$

and

16 $$\begin{aligned} \cos \theta =1-\frac{\theta ^{2}}{2!}+ \frac{\theta ^{4}}{4!}+\cdots . \end{aligned}$$

Substituting (15) and (16) into (13), we have

17 $$\begin{aligned} \frac{d^{2}\theta }{dt^{2}}-(-1+\Lambda )\theta +\frac{1}{6}(-2+2\Lambda )\theta ^{3}+ \frac{1}{120}(1-16\Lambda )\theta ^{5}=0. \end{aligned}$$

Given that both problems (11) and (17) can be viewed as highly nonlinear in nature. We will apply the HPM, LSHPM and the MATLAB built in function bvp5c to solve these problems.

## Implementation of LSHPM

Now, we proceed to address problems (11) and (17) with the help of examples by varying the parameter values.

### Example 4.1

By taking $K = 100 \ Newton/meter^{2}$ , $g = 9.8 meter/sec^{2}$, $m_{1} = 5Kg$ $m_{2} = 1Kg$, $L = 1meter$, $A = \frac{\pi }{6} meter$, we rewrite the problem (11) as (11) as

18 $$\begin{aligned}{} & {} {\frac{{\textrm{d}}^{2}}{{\textrm{d}}{t}^{2}}}v +\frac{1}{5} v^{2} {\frac{{\textrm{d}}^{2}}{{\textrm{d}}{t}^{2}}}v+\frac{1}{5} v \left( {\frac{\textrm{d}}{{\textrm{d}}t}}v \right) ^{2}+{ \frac{549}{25}}\,v +{\frac{49}{50}}v^{3}=0, \end{aligned}$$

19 $$\begin{aligned}{} & {} v(0)=\frac{\pi }{6}, \ \ \ \frac{dv}{dt}|_{t=0}=0. \end{aligned}$$

As previously noted, the initial step in addressing equation (18) using the HPM method involves introducing a constant *p* as the perturbation factor, which is then integrated into Eq. (18). This results in the formulation of the resultant homotopy equation:

20 $$\begin{aligned} H(v, p) = \left\{ \begin{array}{ll} (1-p)\left( {\frac{{\textrm{d}}^{2}}{{\textrm{d}}{t}^{2}}}v + {\frac{549}{25}}\,v \right) + p\left( {\frac{{\textrm{d}}^{2}}{{\textrm{d}}{t}^{2}}}v +\frac{1}{5} v^{2} {\frac{{\textrm{d}}^{2}}{{\textrm{d}}{t}^{2}}}v+\frac{1}{5} v \left( {\frac{\textrm{d}}{{\textrm{d}}t}}v \right) ^{2}+{ \frac{549}{25}}\,v +{\frac{49}{50}}v^{3}\right) =0. \end{array} \right. \end{aligned}$$

Upon substituting Eq. (7) into Eq. (20) and conducting simplifications and rearrangements that are contingent on the powers of *p*, we can derive the corresponding parameters

21 $$\begin{aligned}{} & {} p^{0}: \left\{ \begin{array}{ll} {\frac{{\textrm{d}}^{2}}{{\textrm{d}}{t}^{2}}}v_{{0}} +{\frac{549}{25}\,v_{{0}} }=0, \end{array} \right. \end{aligned}$$

22 $$\begin{aligned}{} & {} p^{1}: \left\{ \begin{array}{ll} {\frac{{\textrm{d}}^{2}}{{\textrm{d}}{t}^{2}}}v_{{1}} +{\frac{549}{25}\,v_{{1}} }+\frac{1}{5}\left( {\frac{{\textrm{d}}^{2} }{{\textrm{d}}{t}^{2}}}v_{{0}} \right) v_{{0}} ^{2}+\frac{1}{5}\,v_{{0}} \left( {\frac{\textrm{d}}{{\textrm{d}}t}}v_{{0}} \right) ^{2}+{ \frac{49}{50}\, v_{{0}} ^{3}}=0, \end{array} \right. \end{aligned}$$

23 $$\begin{aligned}{} & {} p^{2}: \left\{ \begin{array}{ll} {\frac{{\textrm{d}}^{2}}{{\textrm{d}}{t}^{2}}}v_{{2}} +{\frac{549}{25}\,v_{{2}} }+\frac{2}{5}\, \left( {\frac{{\textrm{d}}^{2}}{{\textrm{d}}{t}^{2}}}v_{{0}} \right) v_{{0}} v_{{1}} +\frac{1}{5}\, \left( {\frac{{\textrm{d}}^{2}}{{\textrm{d}}{t}^{2}}}v_{{1}} \right) v_{{0}} ^{2}+ \frac{2}{5}\,v_{{0}} \left( {\frac{\textrm{d}}{{\textrm{d}}t}}v_{{0}} \right) {\frac{\textrm{d}}{{\textrm{d}}t}}v_{{1}} +\frac{1}{5}\,v_{{1}} \left( {\frac{\textrm{d}}{{\textrm{d}}t}}v_{{0}}\right) ^{2}+\\ {\frac{147}{50}\, v_{{0}} ^{2}v_{{1}} }=0, \end{array} \right. \end{aligned}$$

$$\vdots$$

By solving Eqs. (21)–(23) with appropriate initial conditions, we can ascertain the parameters associated with $v_{i}$ for $i=0, 1, 2, \ldots$. It is noteworthy that, except for Eq. (21), the remaining equations are addressed with a zero initial condition. Upon resolving Eqs. (21) through (23), we can determine the values for $v_{0}$, $v_{1}$, and $v_{2}$. Through the synthesis of the solutions to Eqs. (21) through (23), we obtain the approximate solution for Eq. (18) using the HPM in the following manner.

$$\begin{aligned} v=v_{0}+v_{1}+v_{2}. \end{aligned}$$

This implies

24 $$\begin{aligned} v&= 0.0002524761914\, \cos ^{5}(\alpha t)- 0.006546499663\, \cos ^{3}(\alpha t)+ 0.5298927993\,\cos \left( \alpha \,t \right) - \nonumber \\&\quad 0.0008175481825\, t \cos ^{2}(\alpha t)\sin \left( \alpha t \right) + 0.02200129925\,t \sin \left( \alpha \,t \right) - 0.0004781577738 \,{t}^{2} \cos \left( \alpha t \right) . \end{aligned}$$

It is crucial to note that incorporating a greater number of components, such as $v_{3}$, $v_{4}$, and so forth, within *v* in Eq. (24) has the potential to further enhance accuracy and minimize errors to a greater extent. Subsequently, Eq. (24) comprises various terms, including $\cos ^5(\alpha t)$, $\cos ^3(\alpha t)$, $\cos (\alpha t)$, $t\cos ^2(\alpha t)\sin (\alpha t)$, $t\sin (\alpha t)$, and $t^2\cos (\alpha t)$, where $\alpha =4.686149806$. With this information, we propose that our trial solution for Eq. (18) within the LSHPM framework takes the following form:

25 $$\begin{aligned} {\tilde{v}} = c_{0}\, \cos ^{5}(\alpha t)+c_{1}\, \cos ^{3}(\alpha t)+ c_{2}\,\cos \left( \alpha \,t \right) + c_{3}\, t \cos ^{2}(\alpha t)\sin \left( \alpha t \right) + c_{4}\,t \sin \left( \alpha \,t \right) +c_{5} \,{t}^{2} \cos \left( \alpha \,t \right) . \end{aligned}$$

When we apply the boundary conditions (19) to Eq. (25), we obtain

26 $$\begin{aligned} c_{0}= \pi /6-c_{{1}}-c_{{2}}. \end{aligned}$$

By putting (26) in (25), we have

27 $$\begin{aligned} {\tilde{v}} = \left( \frac{\pi }{6}-c_{{1}}-c_{{2}} \right) \cos ^{5}(\alpha t) +c_{{1}} \cos ^{3}(\alpha t)+c_{{2}}\cos \left( \alpha \,t \right) + c_{{3}} \cos ^{2}(\alpha t)\sin \left( \alpha \,t \right) t+c_{{4}} t \sin \left( \alpha \,t \right) + c_{{5}} {t}^{2} \cos \left( \alpha \,t \right) . \end{aligned}$$

We establish the residual function by substituting ${\tilde{v}}$ in place of *v* in Eq. (18), we have

28 $$\begin{aligned} {\hat{R}}(t, c_{1}, c_{2}, c_{3}, c_{4}, c_{5})={\frac{{\textrm{d}}^{2}}{{\textrm{d}}{t}^{2}}}{\tilde{v}}+\frac{1}{5}\, \left( {\frac{{\textrm{d}}^{2}}{{\textrm{d}}{t}^{2}}}{\tilde{v}} \right) \left( {\tilde{v}} \right) ^{2}+\frac{1}{5}{\tilde{v}} \left( {\frac{\textrm{d}}{{\textrm{d}}t}}{\tilde{v}} \left( t \right) \right) ^{2}+{ \frac{549}{25}\,{\tilde{v}} }+{\frac{49}{50}\, \left( {\tilde{v}} \right) ^{3}}. \end{aligned}$$

Upon performing the computation represented by Eq. (29):

29 $$\begin{aligned} J(c_{1}, c_{2}, c_{3}, c_{4}, c_{5})=\int _{0}^{3} {\hat{R}}^{2}(t, c_{1}, c_{2}, c_{3}, c_{4}, c_{5}),dt, \end{aligned}$$

The optimal values for the variables $c_{i}$, with *i* ranging from 1 to 5, can be obtained by calculating the partial derivatives $\displaystyle \frac{\partial J}{\partial c_{i}}$ using MAPLE 2016 software. Once these optimal values are determined, they can be substituted into the proposed solution as outlined in Eq. (25):

30 $$\begin{aligned} {\tilde{v}}&= 0.0002205067\, \left( \cos \left( 4.686149806 t\right) \right) ^{5}- 0.006357987814\, \left( \cos \left( 4.686149806 t \right) \right) ^{3}+ \nonumber \\&\quad 0.5297362569\,\cos \left( 4.686149806 t \right) - 0.0007420527380\, \left( \cos \left( 4.686149806 t \right) \right) ^{2}\sin \left( 4.686149806 t \right) t+ \nonumber \\&\quad 0.02190612201\,\sin \left( 4.686149806 t \right) t- 0.0004639409468\,\cos \left( 4.686149806 t \right) {t}^{2}. \end{aligned}$$

**Table 1:** Comparison of HPM, LSHPM, AGM and bvp5c results when $k = 100$, $g = 9.8$, $m_{1} = 5,$ $m_{2} = 1$, $L = 1$, $A = \frac{\pi }{6}$.

t	bvp5c	AGM^16^	HPM	LSHPM	$|AGM-bvp5c|$	$|HPM-bvp5c|$	$|LSHPM-bvp5c|$
0	0.52359877	0.52359878	0.5235988	0.52359877	0	0	0
0.3	0.09348159	0.10071564	0.0934789	0.09342673	$7.23\times 10^{-3}$	$2.62\times 10^{-6}$	$5.48\times 10^{-5}$
0.6	− 0.49167124	− 0.48485292	− 0.4916685	− 0.49166608	$6.82\times 10^{-3}$	$2.69\times 10^{-6}$	$5.15\times 10^{-6}$
0.9	− 0.26805498	− 0.28724119	− 0.2680486	− 0.26793718	$1.91\times 10^{-2}$	$6.40\times 10^{-6}$	$1.17\times 10^{-4}$
1.2	0.39917946	0.37434968	0.3991713	0.39918235	$2.48\times 10^{-2}$	$8.21\times 10^{-6}$	$2.88\times 10^{-6}$
1.5	0.40780643	0.43125554	0.4077659	0.40768673	$2.34\times 10^{-2}$	$4.06\times 10^{-5}$	$1.19\times 10^{-4}$
1.8	− 0.25623998	− 0.20844333	− 0.2562237	− 0.25630187	$4.78\times 10^{-2}$	$1.62\times 10^{-5}$	$6.18\times 10^{-5}$
2.1	− 0.49613197	− 0.51144482	− 0.4959898	− 0.49600062	$1.53\times 10^{-2}$	$1.42\times 10^{-4}$	$1.31\times 10^{-4}$
2.4	0.07994805	0.01168773	0.0798925	0.08008753	$6.83\times 10^{-2}$	$5.55\times 10^{-5}$	$1.39\times 10^{-4}$
2.7	0.52342702	0.51594116	0.5231526	0.52325033	$7.49\times 10^{-3}$	$2.74\times 10^{-4}$	$1.76\times 10^{-4}$
3.0	0.10694792	0.18679763	0.1071116	0.10682589	$7.98\times 10^{-2}$	$1.63\times 10^{-4}$	$1.22\times 10^{-4}$

### Discussion of results of Example 4.1

**Figure 3:**
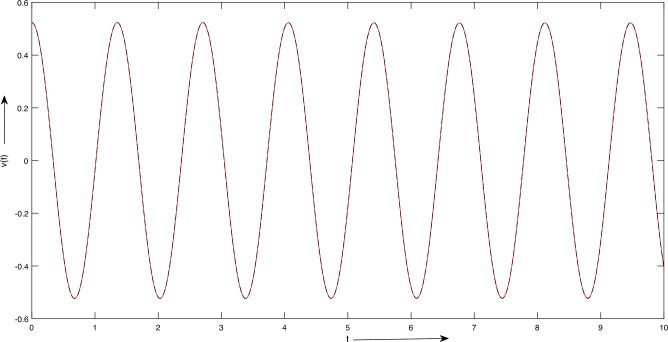
Case 1: Graph representation of solutions Example 4.1.

The numerical results for both the HPM (22) and the LSHPM (28) are summarized in Table 1, and graphical representations of the HPM solution (22) and the LSHPM solution (28) are presented in Fig. 3.

The problem represented by Eqs. (18) and (19) was numerically solved using the bvp5c function in MATLAB, as detailed in references^55^. The analysis revealed that the computed maximum error was approximately $9.779 \times 10^{-11}$.

A comprehensive examination of the results in Table 1 unequivocally demonstrates the superior performance of the LSHPM over AGM^16^. The LSHPM consistently delivers uniform results throughout the entire domain. In stark contrast, the HPM and AGM methods exhibit diminishing convergence as one moves away from the initial point, as illustrated by the data at $x = 2.7$ and $x = 3$ in Table 1.

The numerical solution of the problem represented by Eqs. (18) and (19) was carried out using MATLAB’s bvp5c function, as detailed in references^55^. The computed maximum error was found to be on the order of $9.779 \times 10^{-11}$.

Analysis of the results presented in Table 1 clearly indicates the superior performance of the LSHPM over the AGM^16^. LSHPM consistently delivers uniform results throughout the entire domain, in stark contrast to the HPM and AGM, which exhibits diminishing convergence as one moves away from the initial point, as illustrated by the data at $x = 2.7$ and $x = 3$ in Table 1.

#### Example 4.2

By taking $A = 0.25$, $\Lambda = \frac{\pi }{3}$, we rewrite the problem (17) as

31 $$\begin{aligned}{} & {} {\frac{{\textrm{d}}^{2}}{{\textrm{d}}{t}^{2}}}\theta + \frac{3}{4}\,\theta -\frac{1}{40}\, \theta ^{5}=0, \end{aligned}$$

32 $$\begin{aligned}{} & {} \theta (0)= A, \ \ \ \frac{d\theta }{dt}|_{t=0}=0. \end{aligned}$$

Initially, we formulate a homotopy for Eq. (31) as follows

33 $$\begin{aligned} H(\theta , p) = \left( 1-p \right) \left( {\frac{{\textrm{d}}^{2}}{{\textrm{d}}{t}^{2}}} \theta +\frac{3}{4}\,\theta \right) +p \left( {\frac{{\textrm{d}}^{2}}{{\textrm{d}}{t}^{2}}}\theta + \frac{3}{4}\,\theta -\frac{1}{40}\, \theta \right) ^{5} =0. \end{aligned}$$

After substituting $\theta = \theta _{0}+p\theta _{1}+p^{2}v_{2}+p^{3}\theta _{3}+\cdots$ into Eq. (33) and performing some simplification and rearrangement based on the powers of *p*, we have the following system of differential equations

34 $$\begin{aligned}{} & {} p^{0}: \left\{ \begin{array}{ll} {\frac{{\textrm{d}}^{2}}{{\textrm{d}}{t}^{2}}}\theta _{{0}} +\frac{3}{4}\,\theta _{{0}} =0, \end{array} \right. \end{aligned}$$

35 $$\begin{aligned}{} & {} p^{1}: \left\{ \begin{array}{ll} {\frac{{\textrm{d}}^{2}}{{\textrm{d}}{t}^{2}}}\theta _{{1}} +\frac{3}{4}\,\theta _{{1}} -\frac{1}{40}\, \theta _{{0}} ^{5}=0, \end{array} \right. \end{aligned}$$

36 $$\begin{aligned}{} & {} p^{2}: \left\{ \begin{array}{ll} {\frac{{\textrm{d}}^{2}}{{\textrm{d}}{x}^{2}}}\theta _{{2}} + 0.75\,\theta _{{2}} - 0.1250000000\,\theta _{{1}} \theta _{{0}} ^{4}=0, \end{array} \right. \end{aligned}$$

$$\vdots$$

By solving equations (34) through (36) and applying the appropriate initial conditions, we can determine the parameters $\theta _{0}$, $\theta _{1}$, and $\theta _{2}$. It is essential to emphasize that, apart from Eq. (34), the remaining equations are solved assuming zero initial conditions. Combining the solutions from Eqs. (34)–(36), we can derive an approximate HPM solution for Eq. (31), expressed as follows:

$$\begin{aligned} \theta =\theta _{0}+\theta _{1}+\theta _{2}. \end{aligned}$$

This implies

37 $$\begin{aligned} \theta&= \, 0.01152903607\,t \sin \left( {\hat{\alpha }} \,t \right) + 1.053490429\,\cos \left( {\hat{\alpha }}\,t \right) - 0.00006162350038\,{t}^{2}\cos \left( {\hat{\alpha }}\,t \right) - \nonumber \\&\quad 0.0001423133782\,t \sin \left( {\hat{\alpha }}\,t \right) \cos ^{2}(\alpha t)- 0.004519692720\, \cos ^{3}(\alpha t)- \nonumber \\&\quad 0.00009487558549\, t \sin \left( {\hat{\alpha }}\,t \right) \cos ^{4}(\alpha t)- 0.001802399444\, \cos ^{5}(\alpha t)+ \nonumber \\&\quad 0.00002483198831\, \cos ^{7}(\alpha t)+ 0.000004382115584\, \cos ^{9}(\alpha t), \end{aligned}$$

where ${\hat{\alpha }}=0.866025404$ and $\alpha =4.686149806$. It follows that (37) consist of $t \sin \left( {\hat{\alpha }}\,t \right)$ ,

$$\cos \left( {\hat{\alpha }}\,t \right) , \ {t}^{2}\cos \left( {\hat{\alpha }}\,t \right) , \ t \sin \left( {\hat{\alpha }}\,t \right) \cos ^{2}(\alpha t),$$ $$\cos ^{3}(\alpha t), \ t \sin \left( {\hat{\alpha }}\,t \right) \cos ^{4}(\alpha t), \ \cos ^{5}(\alpha t), \ \cos ^{7}(\alpha t), \ \cos ^{9}(\alpha t)$$ .

With this information, we propose that our trial solution for Eq. (31) within the LSHPM framework takes the following form

38 $$\begin{aligned} {\tilde{\theta }}&= c_{{0}} \cos ^{9}(\alpha t)+c_{{1}} \cos ^{7}(\alpha t)+c_{{2}} \cos ^{5}(\alpha t)+ c_{{3}}t \sin \left( {\hat{\alpha }}\,t \right) \cos ^{4}(\alpha t)\nonumber \\&\quad + c_{{4}} \cos ^{3}(\alpha t)+c_{{5}}t \sin \left( {\hat{\alpha }}\,t \right) \cos ^{2}(\alpha t)+c _{{6}}\cos \left( {\hat{\alpha }}\,t \right) +c_{{7}}\cos ^{2}(\alpha t)+c_{{8}}\sin \left( {\hat{\alpha }}\,t \right) t. \end{aligned}$$

Replacing ${\tilde{\theta }}$ for $\theta$ in Eq. (32), and applying the boundary conditions described in (32) through (38), we derive the following results

39 $$\begin{aligned} c_{{0}}= \frac{\pi }{3}-c_{{1}}-c_{{2}}-c_{{4}}-c_{{6}}. \end{aligned}$$

Substituting (39) in (38), we have

$$\begin{aligned} {\tilde{\theta }}&= (\frac{\pi }{3}-c_{{1}}-c_{{2}}-c_{{4}}-c_{{6}}) \cos ^{9}(\alpha t)+c_{{1}} \cos ^{7}(\alpha t)+c_{{2}} \cos ^{5}(\alpha t)+ c_{{3}}t \sin \left( {\hat{\alpha }},t \right) \cos ^{4}(\alpha t) \\&\quad + c_{{4}} \cos ^{3}(\alpha t)+c_{{5}}t \sin \left( {\hat{\alpha }}\,t \right) \cos ^{2}(\alpha t)+ c_{{6}}\cos \left( {\hat{\alpha }}\,t \right) +c_{{7}}\cos \left( {\hat{\alpha }}\,t \right) {t}^{2}+c_{{8}}\sin \left( {\hat{\alpha }}\,t \right) t. \end{aligned}$$

Replacing ${\tilde{\theta }}$ for $\theta$ in Eq. (31),We establish the residual function as

40 $$\begin{aligned} {\hat{R}}(t, c_{1}, c_{2}, c_{3}, c_{4}, c_{5}, c_{6}, c_{7}, c_{8})={\frac{{\textrm{d}}^{2}}{{\textrm{d}}{t}^{2}}}{\tilde{\theta }} \left( t \right) + 3/4\,{\tilde{\theta }} \left( t \right) -1/40\, \left( {\tilde{\theta }} \left( t \right) \right) ^{5}. \end{aligned}$$

The error associated with the squared residual function is calculated as

41 $$\begin{aligned} J(c_{1}, c_{2}, c_{3}, c_{4}, c_{5}, c_{6}, c_{7}, c_{8})=\int _{0}^{3} {\hat{R}}^{2} (t, c_{1}, c_{2}, c_{3},c_{4}, c_{5}, c_{6}, c_{7}, c_{8})\,dt. \end{aligned}$$

**Figure 4:**
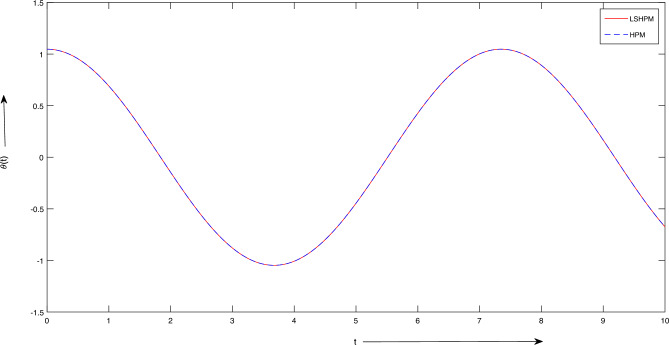
Graph of solutions of Example 4.2.

The optimal values for the variables represented as $c_{i}$ (with *i* ranging from 1 to 8) can be determined by calculating the partial derivatives $\frac{\partial J}{\partial c_{i}}$ (provided in equation 41) using the software MAPLE 2016. Once these optimal values are identified, they can be substituted into the proposed solution as outlined in Eq. (38). Thus, we obtain:

42 $$\begin{aligned} {\tilde{\theta }}(t)&= 0.000002040196957\, \left( \cos \left( {\hat{\alpha }}\,t \right) \right) ^{9}+ 0.00002558107881\, \left( \cos \left( {\hat{\alpha }}\,t \right) \right) ^{7}- \nonumber \\&\quad 0.001795429257\, \left( \cos \left( {\hat{\alpha }}\,t \right) \right) ^{5}- 0.00009499027043\,\sin \left( {\hat{\alpha }}\,t \right) \left( \cos \left( {\hat{\alpha }}\,t \right) \right) ^{4}t -\nonumber \\&\quad 0.004520492279\, \left( \cos \left( {\hat{\alpha }}\,t \right) \right) ^{3}- 0.0001411065420\,\sin \left( {\hat{\alpha }}\,t \right) \left( \cos \left( {\hat{\alpha }}\,t \right) \right) ^{2}t+ \nonumber \\&\quad \left( 1.053485851- 0.00006121848538\,{t}^{2} \right) \cos \left( {\hat{\alpha }}\,t \right) + 0.01153294833\,\sin \left( {\hat{\alpha }}\,t \right) t, \end{aligned}$$

where ${\hat{\alpha }}=0.866025404$. The graphical representations of the HPM solution (37) and the LSHPM solution (41) are illustrated in Fig. 4, and the numerical results for both the above mentioned HPM solution are presented in the Table 2 below

**Table 2:** Comparison of HPM, LSHPM, AGM and bvp5c results when $A = 0.25$, $\Lambda = \frac{\pi }{3}$.

t	bvp5c	AGM^16^	HPM	LSHPM	$|AGM-bvp5c|$	$|HPM-bvp5c|$	$|LSHPM-bvp5c|$
0	1.047197551196598	1.0471975	1.047197550939894	1.047197551196598	$5.12\times 10^{-8}$	0	0
0.3	1.013424671727360	1.013424	1.013424669235789	1.013424683416030	$6.71\times 10^{-7}$	$2.23\times 10^{-9}$	$1.17\times 10^{-8}$
0.6	0.913974676081452	0.91393499	0.913974568049639	0.913974673027138	$3.97\times 10^{-5}$	$1.08\times 10^{-7}$	$3.08\times 10^{-9}$
0.9	0.754613093042295	0.75422333	0.754612307207641	0.754613068460900	$3.89\times 10^{-4}$	$7.86\times 10^{-7}$	$2.47\times 10^{-8}$
1.2	0.545187482426038	0.54340399	0.545185016157168	0.545187481834252	$1.78\times 10^{-3}$	$2.47\times 10^{-6}$	$6.15\times 10^{-10}$
1.5	0.299304758127733	0.29405221	0.299299962043787	0.299304750241314	$5.25\times 10^{-3}$	$4.79\times 10^{-6}$	$7.94\times 10^{-9}$
1.8	0.033344937498370	0.02180802	0.033337895380757	0.033344898483015	$1.15\times 10^{-2}$	$7.04\times 10^{-6}$	$3.91\times 10^{-8}$
2.1	− 0.234852897025061	− 0.25534761	− 0.234861718041070	− 0.234852915665731	$2.05\times 10^{-2}$	$8.82\times 10^{-6}$	$1.87\times 10^{-8}$
2.4	− 0.487292693627270	− 0.51818573	− 0.487302480151352	− 0.487292697296632	$3.09\times 10^{-2}$	$9.79\times 10^{-6}$	$3.72\times 10^{-9}$
2.7	− 0.707109264825293	− 0.74765086	− 0.707118292264257	− 0.707109310645214	$4.05\times 10^{-2}$	$9.03\times 10^{-6}$	$4.59\times 10^{-8}$
3.0	− 0.879899013158141	− 0.92648305	− 0.879904794111942	− 0.879899025013068	$4.66\times 10^{-2}$	$5.78\times 10^{-6}$	$1.19\times 10^{-8}$

### Discussion of results of Example 4.2

The numerical solution for the problem described by Eqs. (31) and (32) was carried out using MATLAB’s bvp5c functionality, with reference to^56^. The calculated maximum error was determined to be approximately $2.203 \times 10^{-14}$. A comprehensive assessment of the outcomes outlined in Table 2 unmistakably demonstrates the superior performance of the LSHPM over AGM^16^ and the Homotopy Perturbation Method (HPM). LSHPM consistently yields uniform results across the entire domain, in stark contrast to AGM and HPM, which display diminishing convergence as one moves away from the initial point, as evident in the data from $x = 1.2$ to $x = 3$ in Table 2.

**Figure 5:**
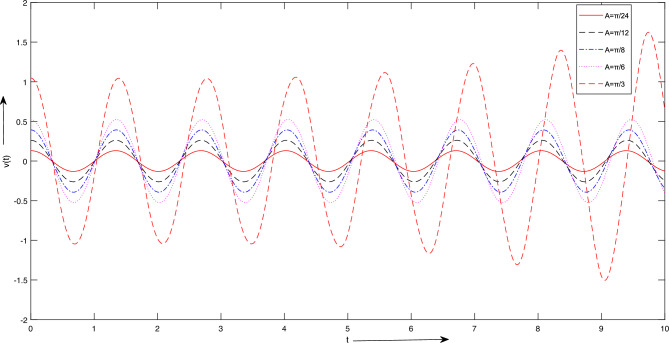
Case 1. Graph for LSHPM solutions with different inititial conditions *A*.

**Figure 6:**
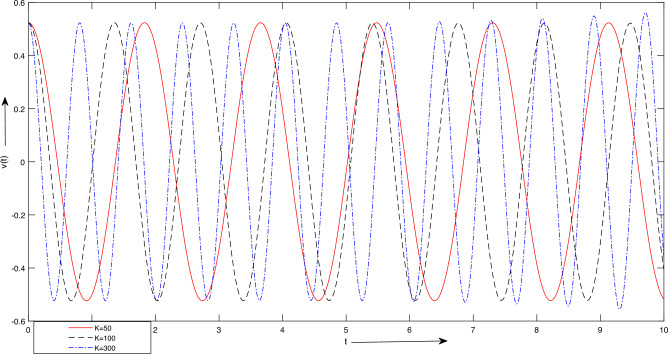
Case 1. Graph for LSHPM solutions with different values of *K*.

**Figure 7:**
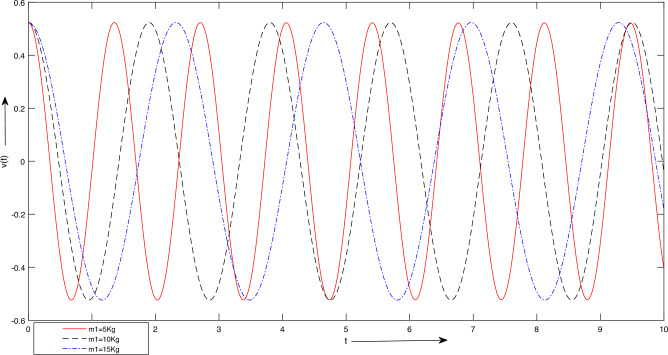
Case 1. Graph for LSHPM solutions with different values of $m_{1}$.

**Figure 8:**
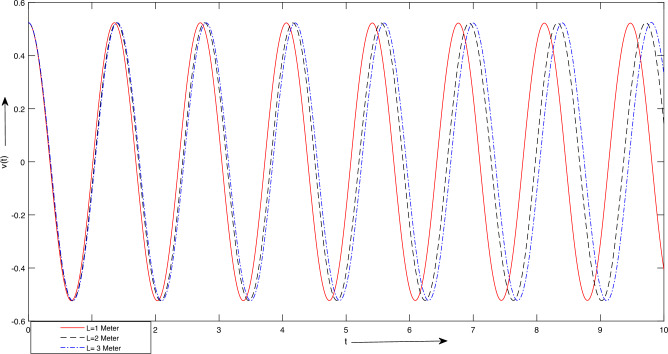
Case 1. Graph for LSHPM solutions with different values of *l*.

**Figure 9:**
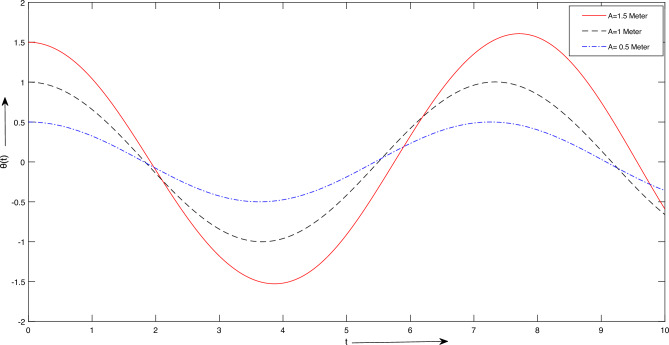
Case 2. Graph for different initial conditions *A*.

**Figure 10:**
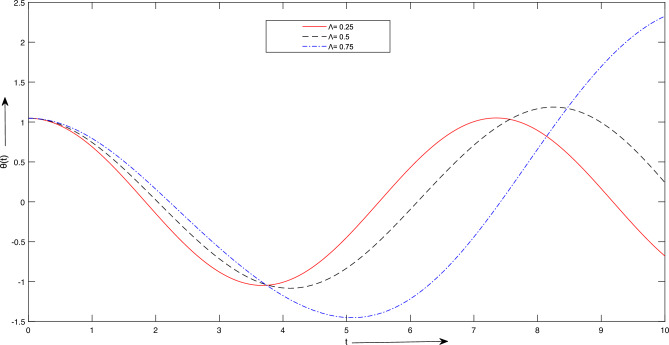
Case 2. Graph of solutions for different values of $\Lambda$.

## Analysis of results

It is worth noting that we utilized software, specifically MAPLE 2016, to solve Example 4.1 and Example 4.2. The computational time for these examples was remarkably efficient, taking only 10 seconds for the former and 3166 seconds for the latter. In Eqs. (29) and (41), we employed Riemann sums with 250 partitions to approximate the integrals.

### Validation

We have validated the three methods, HPM, LSHPM, and the numerical method using MATLAB’s builtin function bvp5c for both Examples as illustrated in Table 1 and Table 2. As shown in these tables, it is clear that HPM and LSHPM work better and are more accurate than AGM^16^ in this investigation. In “Analysis of results” section, we provide a detailed description of the initial conditions for both scenarios. As this study primarily aims to assess the influence of individual parameters on the system’s response, we employ LSHPM with a variety of initial conditions to examine the results. Next, we delve into the analysis of Case 1.

The results presented for case 1:

- For Fig. 5, we take $K = 100 Newton/meter^{2}$, $g = 9.8 \ meter/sec^{2}$, $m_{1} = 5 \ Kg$ $m_{2} = 1\ Kg$, $L = 1\ meter$, $A =[ \frac{\pi }{24},\ \frac{\pi }{12}, \ \frac{\pi }{8},\ \frac{\pi }{6},\ \frac{\pi }{3}] meter$. Figure 5 reveal that changes in amplitude have no discernible impact on the period or frequency; nonetheless, they exert a significant influence on the system’s behavior. In oscillatory systems, the count and regularity of oscillations are fundamental attributes. It is feasible to scrutinize the motion of the system and ascertain the quantity of oscillations occurring within a defined time frame through the resolution of the equations of motion.
- For Fig. 6, we take the values as: $K = [50, 100, 300] Newton/meter^{2}$, $g = 9.8 \ meter/sec^{2}$, $m_{1} = 5 \ Kg$ $m_{2} = 1\ Kg$, $L = 1\ meter$, $A =\frac{\pi }{6},\ \frac{\pi }{3}] meter$. Figure 6 depicts that as the stiffness increases, there is a noticeable decrease in the duration of the oscillation’s period. Specifically, when the stiffness value is elevated from 50 to 300, the period time diminishes from approximately two seconds to less than one second within a three-second interval.
- For Fig. 7, we take $K = 100 Newton/meter^{2}$, $g = 9.8 \ meter/sec^{2}$, $m_{1} =[ 5,\ 10,\ 15]\ Kg,$ $m_{2} = 1\ Kg$, $L = 1\ meter$, $A =\frac{\pi }{6},\ \frac{\pi }{3}] meter$. The findings in Fig. 7 suggest that an increase in mass results in an extended period of oscillation. This occurs because as the mass of the block increases, the system’s inertia also increases, leading to a reduction in frequency. This behavior closely resembles the observations in Fig. 6, where stiffness modifications produced analogous effects.
- For Fig. 7, we take $K = 100 Newton/meter^{2}$, $g = 9.8 \ meter/sec^{2}$, $m_{1} =5\ Kg,$ $m_{2} = 1\ Kg$, $L = [1,\ 2,\ 3] \ meter$, $A =\frac{\pi }{6},\ \frac{\pi }{3}] meter$. Figure 8 provides insights into the influence of increasing the length parameter, denoted as *L*, on the period of oscillation. It is observed that augmenting the length results in a minor extension of the oscillation period. However, this particular parameter exerts a less pronounced effect on the system’s temporal response and frequency when compared to other variables. Additionally, Fig. 8 illustrates the consequences of altering amplitudes on the system’s temporal behavior. Any adjustments in amplitude manifest as changes in the slope of the curves, signifying an increase in angular velocity. Notably, these alterations in amplitude do not significantly impact the system’s oscillation period.

The results presented for case 2:

- For Fig. 9, we take $\Lambda = \frac{\pi }{3}$, A =[0.5, 1, 1.5]. Figure 9 depicts how various amplitudes impact the time response of the system. It is evident that alterations in amplitude result in a more or less abrupt shift in the slope of the lines, signifying an increase in angular velocity. As shown in Fig. 9, adjustments in amplitude do not significantly influence the system’s period time.
- For Fig. 10, we consider $A = \frac{\pi }{3}$, $\Lambda$ =[0.5, 1, 1.5]. Figure 10 demonstrates the impact of varying $\Lambda$ values on the system’s time response. Once again, a noticeable shift in time response is observed when significant changes occur in the parameter $\Lambda$, as depicted in Fig. 10.

In upcoming endeavors, our approach will involve the utilization of LSHPM alongside the MATLAB built-in function bvp5c for the resolution of diverse mathematical models that encompass nonlinear ordinary, partial, and fractional differential equations.

## Conclusion

In our investigation, we utilized three different approaches-MATLAB’s built-in function bvp5c, HPM, and LSHPM-to explore two distinct oscillating systems, comparing their results against numerical solutions. The comparative error analysis for both cases, as presented in Tables 1 and 2, highlights the effectiveness of these methods in dealing with oscillating systems. Notably, LSHPM demonstrated superior convergence rates and higher accuracy when compared to AGM and HPM, as clearly indicated in Tables 1 and 2.

The adaptability of these methods was demonstrated by modifying individual parameters, such as stiffness or length, showcasing LSHPM’s ability to deliver highly accurate results closely aligned with numerical values. Furthermore, the exploration of various parameters revealed that stiffness significantly influenced the system’s period time, while mass and length had inverse effects. Importantly, variations in length were observed to have a relatively milder impact on the system’s temporal response and frequency when compared to changes in stiffness and mass.

## Data Availability

All data generated or analyzed during this study are included in this published article.
